# Predictive capacity of peritransplant measurable residual disease thresholds in *NPM1*-mutant acute myeloid leukemia

**DOI:** 10.1182/bloodadvances.2025017908

**Published:** 2025-12-04

**Authors:** Jan Christian Schroeder, Friederike Schwartz, Judith Metzdorf, Nick Barensteiner, Lucas Mix, Lisa-Marie Necke, Adrian Fehn, Andreas Riedel, Liv Jentzsch, Philipp Faustmann, Wichard Vogel, Wolfgang Andreas Bethge, Thomas Schroeder, Claudia Lengerke

**Affiliations:** 1 Department for Hematology, Oncology, Clinical Immunology and Rheumatology, University Hospital Tübingen, Tübingen, Germany; 2 Department of Hematology and Stem Cell Transplantation, West German Cancer Center Essen, University Hospital Essen, Essen, Germany; 3 German Cancer Consortium, Partner Site Tübingen, a partnership between DKFZ and University Hospital Tübingen, Tübingen, Germany

## Abstract

Measurable residual disease (MRD) monitoring for mutated *NPM1* is increasingly used to guide treatment decisions in patients with acute myeloid leukemia (AML) carrying this mutation. *NPM1*-MRD positivity after induction has been shown to identify patients who may benefit from allogeneic hematopoietic cell transplantation (allo-HCT), and *NPM1*-MRD monitoring after allo-HCT can detect early relapse, enabling the prompt initiation of salvage therapy. However, recommendations for clinical decision-making based on peritransplant *NPM1*-MRD levels are missing. In this study, we retrospectively analyzed 172 patients with *NPM1*-mutant AML treated at 2 German centers to explore the predictive values of *NPM1*-MRD measured before and after allo-HCT. We found that pretransplant MRD negativity was a strong predictor of favorable long-term overall survival (OS). In contrast, patients with positive and negative *NPM1*-MRD status at day 30 after HCT showed comparable OS. Finally, statistically derived *NPM1*-MRD thresholds effectively stratified MRD-high and MRD-low patient groups with differential outcome, with 2 peritransplant MRD risk scores obtained by longitudinal integration. First, a combined score using MRD measurements before HCT and at day 30 HCT was used to guide early reduction of immunosuppression (concordance index [C-index], 0.737). Second, a combined score using MRD measurements before HCT and at day 30 and day 100 after HCT was used to guide later post-HCT interventions (C-index, 0.841; stratified 2-year OS groups, 100%, 90.1%, 57.1%, and 25.7%; *P* < .0001). This approach predicted OS better than age, FLT3-ITD status, or morphological remission status. We propose that in the peritransplant setting, *NPM1*-MRD thresholds are superior to conventional MRD analysis based on binary or log-step change data.

## Key Points

• *NPM1*-MRD thresholds distinguish MRD-high patients with poor prognosis from MRD-low and MRD-negative patients with favorable outcomes.
• Peritransplant *NPM1*-MRD thresholds are more accurate predictors of outcome than traditional binary or log-change–based MRD interpretation.

## Visual Abstract

**Figure:**
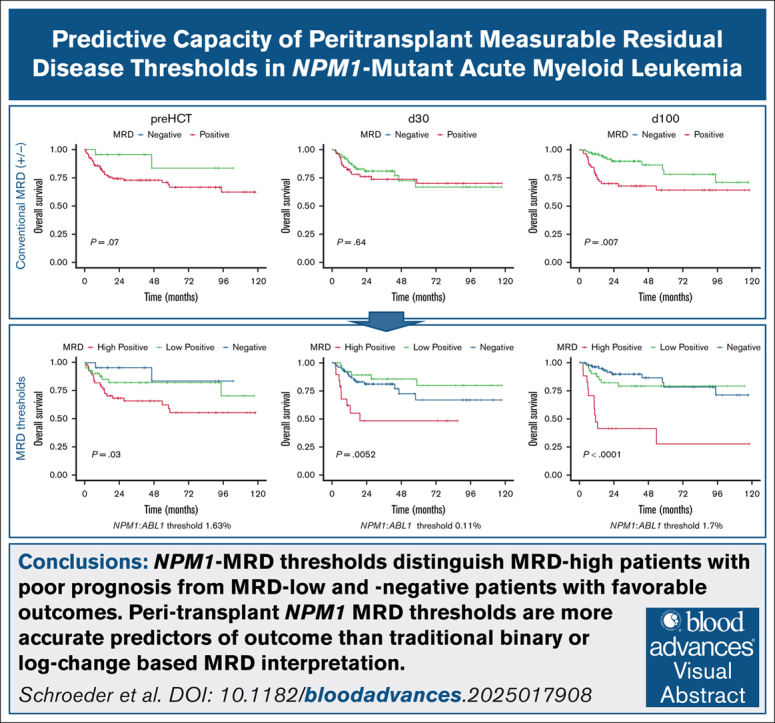

## Introduction

Acute myeloid leukemia with mutated *NPM1* (*NPM1*-mutant AML) is the most common genetic AML subtype. This AML subtype generally affects younger patients and, in the absence of concurrent FLT3-ITD, is considered to have a favorable prognosis.^1^ However, accumulating data suggest patients showing *NPM1* measurable residual disease (MRD) after induction are at an increased risk for relapse and should be allocated to subsequent allogeneic hematopoietic cell transplantation (allo-HCT).^2^^,^^3^ Therefore, the highly sensitive and specific detection of *NPM1*-mutant transcripts with quantitative reverse transcription polymerase chain reaction (qRT-PCR) for molecular MRD monitoring serves as an essential tool to inform prognosis and guide treatment. Most of the available evidence focuses on *NPM1*-MRD assessment after 1 to 2 cycles of induction therapy^4^, ^5^, ^6^, ^7^, ^8^, ^9^, ^10^, ^11^ or before allo-HCT^12^, ^13^, ^14^, ^15^, ^16^, ^17^ (pre-HCT). At reported detection thresholds ranging from 10^˗4^ to 10^˗7^ *NPM1*-mutant transcripts, most studies propose MRD negativity as the optimal cutoff for stratifying patients into favorable vs unfavorable prognostic groups and consequently, for guiding the decision to proceed with allo-HCT. At the pre-HCT time point, optimal thresholds ranging from the detection limit up to *NPM1*:*ABL1* ratios of 1% have been reported, with patients who are *NPM1*-MRD negative or MRD low demonstrating superior outcomes.^18^ For patients treated with AML receiving intensive chemotherapy–based induction and consolidation, the MRD working party of the Eurpoean LeukemiaNet (ELN) has established robust guidelines on the methodology, application, and interpretation of MRD.^19^^,^^20^ These include the definition of low-level *NPM1*-MRD with an allelic ratio of <2%, which may persist for prolonged periods posttreatment but still associates with favorable outcome.

In contrast to these existing data on pre-HCT MRD, the interpretation of peritransplant MRD is lacking such clear recommendations. A recent retrospective analysis did not find a prognostic impact of pre-HCT MRD negativity (below the detection limit) on post-HCT outcomes.^21^ Previous comprehensive analyses have demonstrated an enhanced predictive capacity of pre-HCT MRD when a statistically derived threshold is applied.^14^^,^^22^ For early post-HCT *NPM1*-MRD monitoring, only few studies with small sample sizes and heterogeneous methods of *NPM1*-MRD detection (sequencing vs qRT-PCR based) are available.^23^, ^24^, ^25^ In this study, we comprehensively investigated qRT-PCR–based *NPM1*-MRD trajectories at 3 defined peritransplant time points and explored their association with clinical parameters in a real-world cohort of patients with *NPM1*-mutant AML undergoing allo-HCT.

## Methods

### Patients and treatment

We retrospectively analyzed 172 patients with *NPM1*-mutant AML who consecutively underwent a transplant between 2005 and 2024 at 2 German centers. A total of 115 and 57 patients received allo-HCT at the University Hospital of Tübingen and the University Hospital of Essen, respectively. We included only patients who received intensive chemotherapy as the first-line treatment and did not have adverse genetic features according to the ELN 2017 guidelines and analyzed peritransplant MRD monitoring only for the first allo-HCT of any patient. Most common indications for allo-HCT in eligible patients were relapsed or refractory disease, ELN risk category, and *NPM1*-MRD persistence or relapse. The investigation was conducted according to the Declaration of Helsinki and European data protection regulations and was approved by the institutional review boards of the Eberhard-Karls University of Tübingen (099/2023BO2) and the Medical Faculty of the University Duisburg-Essen (22-10708-BO).

### FLT3 and *NPM1*-MRD assessment

FLT3-ITD status and *NPM1* qRT-PCR MRD measurements were performed as part of routine diagnostics either at the treating institute or at third-party routine diagnostic laboratories. A total of 5 to 10 mL of peripheral blood (PB) or bone marrow (BM) aspirate were subjected to qRT-PCR as described previously by Schnittger et al.^26^ The analysis was performed according to the Europe Against Cancer guidelines, that is, *NPM1* was considered negative at a cycle threshold (CT) value of ≥40 in at least 2 of 3 replicates. MRD was monitored at the following time points: MRD pre-HCT, defined as the MRD measurement nearest to start of conditioning without any additional treatment lines in between; MRD post-HCT day 30, defined as MRD measurement nearest to the time point of 30 days after allo-HCT; and MRD post-HCT day 100, defined as MRD measurement nearest to the time point of 100 days after allo-HCT. MRD relapse or persistence was defined as newly detectable *NPM1*-MRD after the achievement of negativity or newly detected MRD increase of >1 log_10_-step in patients with available MRD measurement at the respective time point.

### Statistical analysis

Descriptive statistics included frequencies and percentages for categorical variables and median, mean, range, and standard deviation for continuous variables. The probabilities of overall survival (OS) and relapse-free survival (RFS) were analyzed using the Kaplan-Meier estimator and log-rank test. Relapse incidence and nonrelapse mortality (NRM) were estimated with relapse and death as competing events using the Gray test. OS was defined as the time from allo-HCT to death, and RFS as the time from allo-HCT to relapse or death, depending on which event occurred first. NRM was defined as death after allo-HCT without prior relapse.

Optimized numerical thresholds to divide prognostic subgroups based on *NPM1*-MRD in PB and/or BM were evaluated in 2 steps. First, maximally selected rank statistics were used to select the numeric threshold with best discriminatory capacity for OS per material and time point. Second, the log-rank test, univariate regression, and area under the receiver operating characteristic curve (AUROC) were used to compare the predictive capacity of MRD thresholds in the different materials PB, BM, or when pooling measurements by using the higher value of one of both per time point. Correlation between PB and BM MRD was assessed using Spearman ρ.

To analyze predictive factors, univariate and multivariate Cox proportional hazards regression were used. In case of nonconverging Cox regression due to small subgroups or perfect separation, Firth correction was applied. Variables for the multivariate models were selected based on plausibility, significance of differences in univariate analysis, and maximization of Akaike information criterion in stepwise variable selection. Prognostic subgroups in multivariate risk models were selected using survival trees. Results are presented as hazard ratio (HR) with 95% confidence interval (CI), with all the tests and CIs being 2-sided. Univariate and multivariate models were validated using bootstrap resampling (200 replicates) with out-of-bag validation. The level of significance was set at 0.05 for all tests. Statistical analysis and visualization of survival and incidence curves were performed using R 4.3.0^27^ and RStudio 2023.03.0.^28^

## Results

### Patient characteristics

A total of 172 patients with *NPM1*-mutant AML were treated with intensive chemotherapy–based induction treatment (7+3) and allo-HCT (Table 1) at the University Hospital Tübingen and the University Hospital Essen were evaluated. In 89 of the 172 patients (51.7%), an FLT3-ITD mutation was codetected. Of these, 50 (56.2%) received midostaurin for induction and/or consolidation, and 36 (40.4%) received FLT3 inhibitors as maintenance (27 patients) or salvage treatment (9 patients) post–allo-HCT. Of the 172 patients, 118 (68.6%) received allo-HCT in first complete remission (CR; CR1), 26 (15.1%) in CR2, and 28 (16.3%) in persistent disease. Of 118 patients who underwent transplant in CR1, 30 (25.4%) had MRD relapse. Seventeen of 115 patients (14.8%) with available data received myeloablative conditioning, 20 (17.4%) received Fludarabine/Amsacrine/Cytarabine- (FLAMSA-)based sequential reduced-intensity conditioning, and 78 (67.8%) received reduced-intensity conditioning (supplemental Table 1). Of these 115 patients, 43 (37.4%) received an allograft from a matched related donor, 53 (46.1%) from a matched unrelated donor, 15 (13.0%) from a mismatched unrelated donor, and 4 (3.5%) from a haploidentical donor.

**Table 1.** **Patient characteristics and treatment variables**

Variable	Stratum	n (%)
Age, y	<40	15 (8.7)
	40-60	86 (50.0)
	≥60	71 (41.3)
Sex	Male	80 (46.5)
	Female	92 (53.5)
Karnofsky performance score	100	39 (22.7)
	≤90	73 (42.4)
	Not available	60 (34.9)
*NPM1* mutation type	A	85 (49.4)
	B	6 (3.5)
	D	10 (5.8)
	Other	5 (2.9)
	Not reported	66 (38.4)
FLT3-ITD	No	83 (48.3)
	Yes	89 (51.7)
Midostaurin during induction/consolidation	No	39 (43.8)
	Yes	50 (56.2)
Post-HCT FLT3 inhibitor treatment	None	53 (59.6)
	Maintenance	27 (30.3)
	Salvage	9 (10.1)
No. of inductions	1	43 (25.0)
	2	72 (41.9)
	Not available	57 (33.1)
Status HCT	CR1	88 (51.2)
	MRD relapse	30 (17.4)
	CR2	26 (15.1)
	No CR	28 (16.3)
Conditioning	RIC	98 (57.0)
	MAC	17 (9.9)
	Not available	57 (33.1)
Donor	Matched related	43 (25.9)
	Matched unrelated	53 (30.8)
	Mismatched unrelated	15 (8.7)
	Haploidentical	4 (2.3)
	Not available	57 (33.1)

MRD-relapse patients were those with prior MRD relapse, defined by an increase of >1 log_10_ step or newly positive MRD after repeated negative measurements.

MAC, myeloablative conditioning; RIC, reduced-intensity conditioning.

### Allo-HCT treatment outcomes

The Kaplan-Meier estimated probabilities for 5-year OS, RFS, NRM, and relapse incidence from allo-HCT were 70.2% (95% CI, 62.3-79.1), 52.5% (95% CI, 44.0-62.7), 14% (95% CI, 8.8-21), and 33% (95% CI, 25-42), respectively (supplemental Figure 1A).

The median follow-up time was 42 months. In univariate regression analysis, higher age, mismatched donor, and, by trend, Karnofsky performance status, and a higher number of consolidations were associated with inferior OS, whereas myeloablative conditioning was associated with superior OS (supplemental Figure 1B; supplemental Table 2). FLT3-ITD status, treatment with FLT3 inhibitors before or after allo-HCT (supplemental Figure 2), sex, and the number of inductions did not show significant univariate effects.

Patients in CR1 after MRD relapse and patients in CR2 did not have inferior outcomes compared with patients who underwent a transplant in CR1 (supplemental Table 2), but transplantation in active disease did show inferior OS (HR, 3.04; 95% CI, 1.58-5.83; *P* = .001). Therefore, we investigated whether peritransplant *NPM1*-MRD may improve outcome prognostication post-HCT management.

### Peritransplant MRD monitoring

Routine qRT-PCR *NPM1*=MRD was measured in PB and/or BM at multiple standardized time points in most patients. MRD measurements were available for 124 of the 172 patients (72.1%) pre-HCT, for 137 patients (79.7%) post-HCT day 30, for 144 patients (83.7%) post-HCT day 100, and for 102 patients (59.3%) at all 3 time points (supplemental Table 3). The reasons for missing data are heterogeneous and include external MRD assessments performed before allo-HCT referral with incomplete documentation, omission of MRD analysis in patients with active disease, and evolving MRD monitoring standards over time. MRD levels were ∼1 log_10_ higher in BM than in PB samples and demonstrated strong correlation between paired values in PB and BM at the pre-HCT (Spearman ρ, 0.83) and the post-HCT day 30 (0.78) and day 100 time points (0.83; supplemental Table 4).

In the real-world peritransplant setting, MRD assessment may be limited to either the PB or BM; however, for some patients, results from both sources are available, necessitating the interpretation of potentially discordant values. Therefore, we compared the predictive capacity of *NPM1*-MRD measured in either PB or BM in the subset of patients with available measurements from both materials (supplemental Table 5). Analysis based on univariate Cox regression and AUROC confirmed that integrating available MRD measurements from PB and BM is viable. In cases of discrepant MRD measurements between PB and BM, using the more elevated value yielded comparably robust discrimination between patients with higher vs lower MRD levels (supplemental Figure 3). In line with the clinical routine, all patients with a positive MRD measurement in PB or BM were classified as positive, and all patients with a measurement in PB or BM above a statistically derived threshold were classified as MRD high.

### Optimized MRD thresholds increase prognostic value of pre-HCT MRD

**Figure 1.**
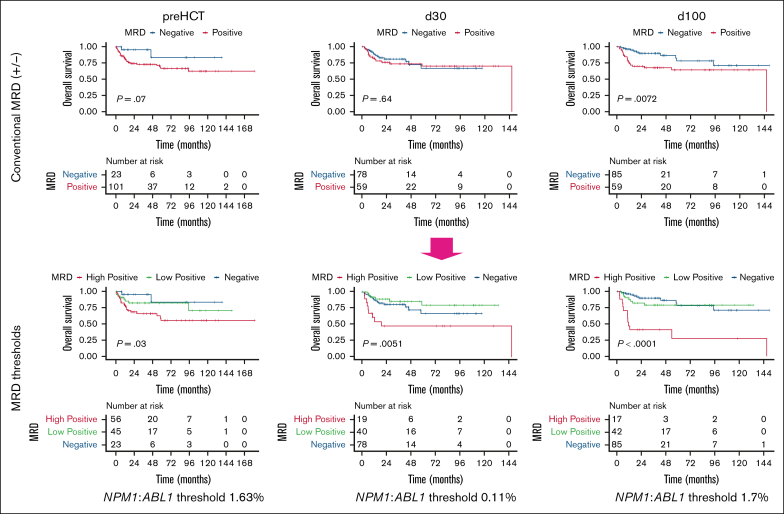
**Optimized thresholds substantially increase predictive capacity of peritransplant MRD.** Comparison of OS stratified by MRD negativity vs the application of statistically derived MRD thresholds at standardized peritransplant time points pre-HCT (n = 124; left), day 30 post-HCT (n = 137; middle), and day 100 post-HCT (n = 144; right) using the Kaplan-Meier estimator and log-rank test.

Pre-HCT MRD negativity was associated with good 5-year OS (83.7% [95% CI, 63.5-100]), but it was detectable in only 23 of the 172 patients (13.4%). Therefore, we hypothesized that a statistically derived optimized MRD threshold might better stratify treatment outcomes and inform prognosis (approach illustrated in supplemental Figure 4). Using maximally selected rank statistics, we identified an optimal pre-HCT cutoff of 1.63% for OS, classifying patients with MRD levels below this threshold into an MRD-low subgroup and those above it into an MRD-high subgroup (Figure 1). Using this stratification, we found that MRD-high patients showed inferior 5-year OS when compared with MRD-low or MRD-negative patients (55.6% vs 82.4% or 83.7%; *P* = .03). Conversely, optimal thresholds to predict relapse and NRM were identified at 0.63% and 83%, respectively (supplemental Figure 5). In contrast, MRD-low patients showed OS comparable with MRD-negative patients.

### Prognostic value of high vs low post-HCT MRD

We next evaluated MRD levels at the time points day 30 and day 100. Interestingly, MRD negativity at day 30 after HCT had no predictive impact on outcome (Figure 1), with day 30 MRD-low vs MRD-negative patients showing comparable 5-year OS. In contrast, the application of an optimized threshold at an *NPM1*:*ABL1* ratio of 0.11% identified a day 30 MRD-high subgroup with higher mortality (high/low/negative: 48.2% vs 79.9% vs 66.9%; *P* = .0052) and an MRD-low subgroup with OS comparable with that of MRD-negative patients (Figure 1). In contrast, MRD negativity at day 100 after HCT strongly enhanced OS. However, an optimized threshold of 1.7% again identified subgroups of MRD-low patients (Figure 1) showing a 5-year OS comparable with that of MRD-negative patients (high/low/negative: 27.5% vs 79.2% vs 78.2%; adjusted *P* < .0001). Although thresholds with predictive capacity for relapse prediction could be derived at day 30 (0.09%) and day 100 (2.25%), there was no significant difference in NRM between MRD-high and -low patients at these time points (supplemental Figure 5).

In summary, thresholding increased the prognostic value of peritransplant MRD monitoring and, importantly, identifies MRD-low patients with similar outcomes compared with that of MRD-negative patients.

### Longitudinal integration of thresholds yields a peritransplant MRD risk score with high predictive accuracy

More than 50% of the patients included in this real-world analysis showed positive MRD measurements after allo-HCT. Therefore, we investigated whether the application of statistically derived thresholds to peritransplant MRD monitoring may improve outcome prediction.

**Figure 2.**
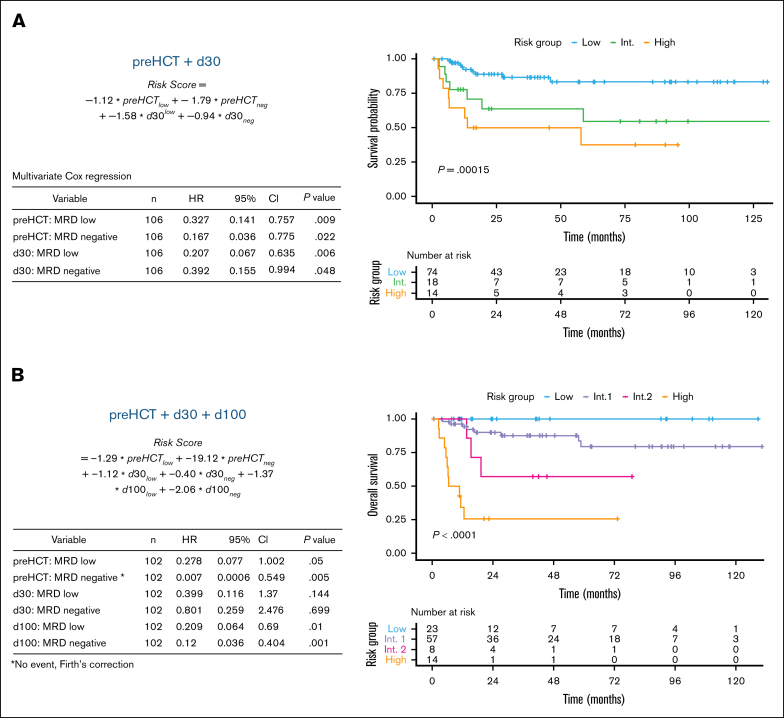
**Peritransplant MRD risk score stratifies post-HCT survival.** Multivariate Cox proportional hazards regression models were fitted using MRD levels stratified into MRD negative, MRD low, and MRD high at peritransplant time points pre-HCT and day 30 only (A) or pre-HCT, day 30, and day 100 (B). In each panel, the risk score calculation based on model coefficients is displayed (top left); HRs, 95% CIs, and multivariate *P* values for constituting MRD time points and strata are displayed in a table (bottom left); optimal stratification of long-term OS into low-, intermediate-, and high-risk groups by the application of the peritransplant MRD risk score using the Kaplan-Meier estimator and log-rank test is displayed (right). Int, intermediate.

To this end, we generated 2 longitudinal Cox proportional hazards regression models for the subgroup of patients with available MRD measurements before and after HCT. To predict outcomes already at day 30 after HCT and guide early interventions, we first generated a longitudinal Cox model based only on the thresholded MRD measurements at pre-HCT and day 30. As shown in Figure 2A, this approach produced a peritransplant MRD risk score by assigning each patient to the MRD-negative, MRD-low, or MRD-high strata at each assessed time point. The score clearly stratified treatment outcomes (concordance index [C-index], 0.737) and outperformed the use of the pre-HCT MRD threshold alone.

We then created a second Cox model using the combined thresholded MRD data from all 3 time points: pre-HCT, day 30, and day 100. The resulting tripartite risk score excellently stratified patient OS, RFS, and relapse rate (supplemental Figure 6) after HCT into 4 strata, with 2-year OS in the low-, intermediate-1–, intermediate-2–, and high-risk groups of 100% (95% CI, 100-100), 90.1% (95% CI, 82.2-98.8), 57.1% (95% CI, 30.1-100), and 25.7% (95% CI, 10.1-65.6), respectively (*P* < .0001; C-index, 0.841). Poor survival in the high-risk group was associated with a high relapse rate of 86.0% after 2 years (supplemental Figure 6), whereas 2-year relapse rates were further stratified in the low, intermediate-1, and intermediate-2 groups (8.3%, 30.0%, and 43.0%, respectively; *P* < .0001).

In contrast, predictions based on relative changes in MRD levels between the peritransplant time points pre-HCT, day 30, and day 100 were insufficient. Although increases of ≥1 log_10_ step between day 30 and day 100 after HCT identified a subgroup of patients with inferior OS, the median relative increases in log_10_ values between day 30 and day 100 were more pronounced in patients without relapse or death after HCT (supplemental Figure 8A). In addition, higher log reductions between pre- and post-HCT time points were paradoxically associated with inferior OS, likely due to bias from high absolute pre-HCT levels (supplemental Figure 8B).

### Analysis of remission subgroups

**Figure 3.**
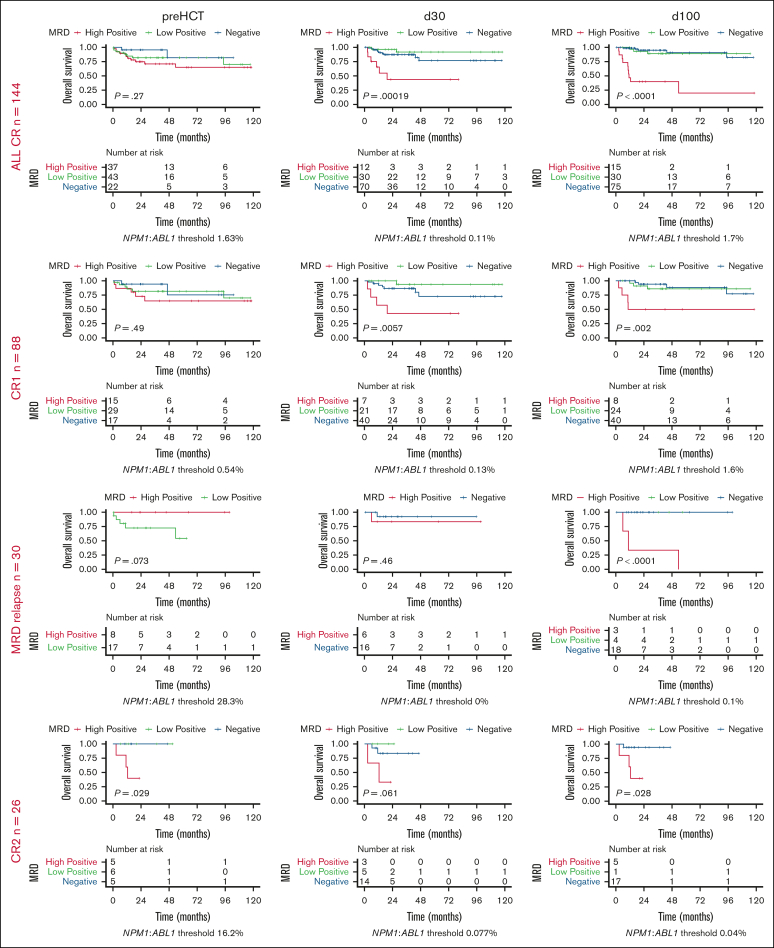

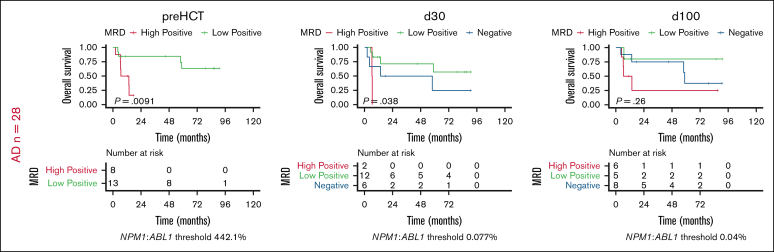
**Predictive capacity of MRD thresholding in subgroups stratified by remission status.** Analysis of OS in the subgroup of patients in CR, CR1, in CR1 but with MRD relapse, in CR2, and with AD before allo-HCT. OS was stratified by the application of statistically derived MRD thresholds to MRD measurements at standardized peritransplant time points pre-HCT (left), day 30 post-HCT (middle), and day 100 post-HCT (right) using the Kaplan-Meier estimator and log-rank test. AD, active disease.

Previous *NPM1*-MRD analyses focused on pre-HCT MRD and excluded patients who underwent a transplant in active disease. We also derived thresholds in patients grouped by remission status (Figure 3). Interestingly, in the subset of 144 patients who received allo-HCT in CR, thresholded pre-HCT MRD was not significantly predictive of OS (*P* = .27). In contrast, MRD thresholds again identified predictive MRD-low and -high subgroups at day 30 (*P* = .00019) and day 100 after HCT (adjusted *P* < .0001), which were superior to distinction into MRD-negative and MRD-positive alone. The loss of the predictive power of pre-HCT MRD, but not post-HCT MRD, was also observed in patients who underwent allo-HCT in CR1 without prior MRD relapse (n = 88). In contrast, among patients in CR1 who had experienced MRD relapse (n = 30), including 4 who had received salvage therapy before conditioning, only the thresholded MRD at day 100 after HCT identified an MRD-high subgroup with significantly inferior OS. In contrast, thresholded pre- and post-HCT MRD identified MRD-high subgroups with inferior OS after allo-HCT in CR2 (n = 26) or active disease (n = 28; Figure 3). The absolute levels of statistically derived MRD thresholds compared with the full cohort were identical in patients who underwent a transplant in CR at all 3 time points and changed only slightly in CR1 patients (pre-HCT lost predictive capacity; day 30, 0.11%-0.13%; day 100, 1.7%-1.6%).

### Further validation

To address overfitting within our data set, we assessed the stability and performance of maximally selected rank MRD cutoffs using bootstrap resampling (200 replicates) with out-of-bag validation (supplemental Table 6). The derived cutoffs were variable pre-HCT (median, 7.6 [interquartile range (IQR), 0.6-79.6]), but more stable at day 30 (median, 0.11 [IQR, 0.08-0.30]), and day 100 (median, 0.83 [IQR, 0.3-1.7]), in line with lacking predictive capacity of pre-HCT MRD in CR patients. Bootstrap resampling of the longitudinal peritransplant MRD risk model showed a median and stable global C-index of 0.783 ± 0.078, providing validation for our approach.

Considering that the thresholds were statistically derived, we also tested and compared a range of clinically usable thresholds at all time points. At all 3 time points, the HRs of MRD-high patients vs MRD-low patients increased with higher MRD thresholds, again confirming an association between higher MRD and inferior outcome. Regarding predictive capacity, the evaluation of AUROC identified optimal thresholds next to the ones derived using maximally selected rank statistics at pre-HCT (2.0%), day 30 (0.1%), and day 100 (2.0%; supplemental Figure 9).

### Additional value of BM MRD assessment

The monitoring of MRD in PB only may spare patients additional invasive BM assessments. Therefore, we investigated whether BM MRD provides additional predictive capacity at peritransplant time points. The head-to-head comparison of MRD in PB vs BM did not suggest higher predictive capacity for MRD measured in BM vs PB at peritransplant time points (supplemental Table 5). Similarly, univariate differences between MRD-high and MRD-low patients were comparable for assessment in PB and BM (supplemental Figure 10).

A pragmatic approach in clinical routine could be to determine MRD in BM only among patients with high or borderline MRD in PB to clearly allocate a patient to the MRD-high or -low groups. However, while patients with high MRD in both PB and BM had clearly inferior outcomes, the predictive capacity of MRD was not increased by including BM values at pre-HCT and day 30 (supplemental Table 4), and our data do not support ruling out of MRD-high status in patients with high MRD in PB but low MRD or negative MRD in BM at any time point (supplemental Figure 10). In contrast, the positive predictive values to find low MRD in BM if low MRD was previously detected in PB were high for all time points (pre-HCT, 97%; day 30, 92.9%; day 100, 95.2%).

### Longitudinal threshold model predicts outcome independent of other clinical variables including FLT3-ITD

Finally, to assess potential confounding, we performed Cox multivariate regression analysis including the peritransplant MRD risk score as well as other patient and treatment variables (Table 2). The peritransplant MRD risk score remained significantly associated with OS (intermediate-1–risk group [HR, 5.11; *P* = .15]; intermediate-2–risk group [HR, 13.91; *P* = .025]; high-risk group [HR, 56.3; *P* < .0001]), as did transplantation in active disease (HR, 1.74; *P* = .042) and higher age (HR, 1.13; *P* = .02). Patients with higher peritransplant MRD risk score were more likely to have other high-risk characteristics, such as older age at allo-HCT, allo-HCT in CR2 or active disease, and FLT3 inhibitor treatment after HCT (supplemental Table 7).

**Table 2.** **Multivariate regression analysis of peritransplant MRD risk score and other patient characteristics and treatment variables**

Variable	Stratum	n	OS, multivariate (Firth correction)	*P* value
			HR	
Risk group	Intermediate-1	102	5.12	.15
	Intermediate-2	102	13.91	.025
	High	102	56.30	<.0001
Age	Cont.	102	1.14	.02
Remission at HCT	No CR	102	1.74	.042

Variables were analyzed for significant univariate or multivariate associations with OS using multivariate Cox regression. The results are shown as HRs and univariate *P* values. Firth correction was applied to the multivariate regression.

Cont., age analyzed as continuous variable.

**Figure 4.**
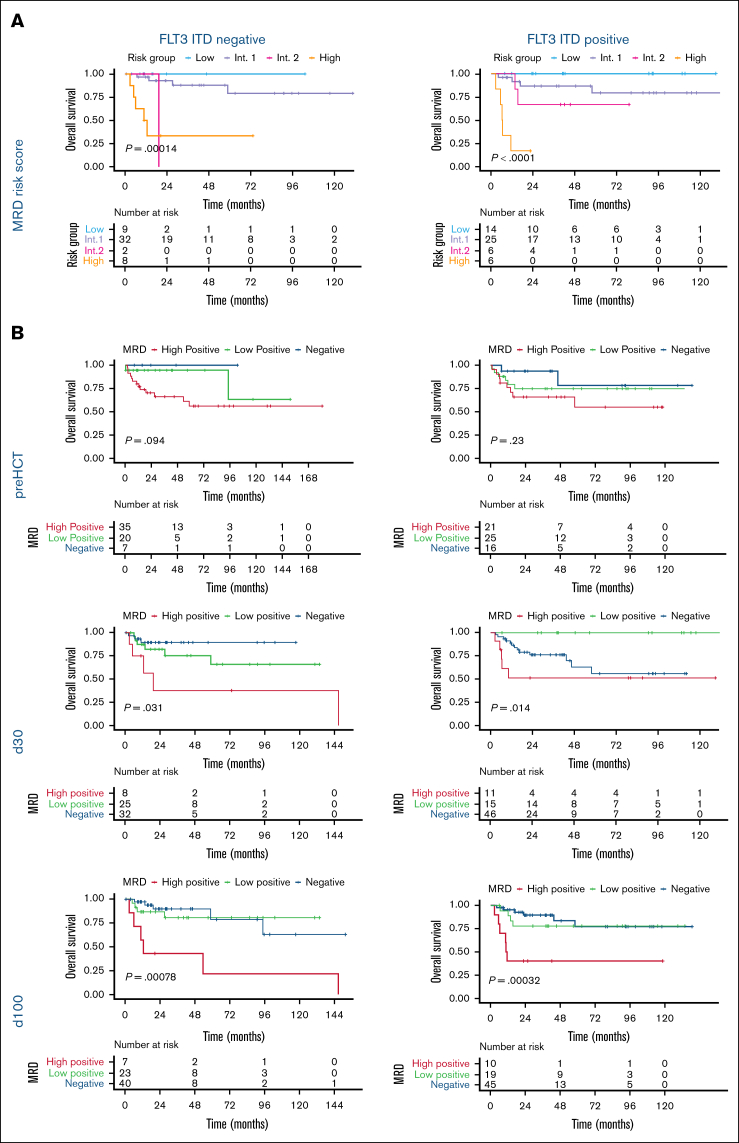
**Peritransplant MRD risk score and thresholds predict survival irrespective of FLT3-ITD status.** (A) Stratification of OS for the subgroups of FLT3-ITD^−^ (left) and FLT3-ITD^+^ (right) patients into low-, intermediate-, and high-risk groups by the application of the longitudinal pre-HCT, day 30, day 100 peritransplant MRD risk score using the Kaplan-Meier estimator and log-rank test. (B) Comparison of OS for both FLT3-ITD^˗^ (left) and FLT3-ITD^+^ (right) patients stratified by MRD negativity and the application of statistically derived MRD thresholds at standardized peritransplant time points pre-HCT (top), day 30 post-HCT (middle), and day 100 post-HCT (bottom) using the Kaplan-Meier estimator and log-rank test. Int, intermediate.

Previous analyses suggested that an *NPM1*=MRD-low status pre-HCT might differentially affect patients depending on FLT3-ITD codetection.^14^ In our cohort, FLT3-ITD status did not influence OS, most likely due to the center referral of FLT3-ITD patients in CR1 to allo-HCT, whereas patients without FLT3-ITD underwent a transplant because of MRD persistence or relapsed or refractory disease. Therefore, we investigated whether FLT3-ITD status alone might still affect stratification of OS according to MRD. However, we observed OS stratification by the longitudinal MRD risk score for both FLT3-ITD^−^ and FLT3-ITD^+^ patients (Figure 4A), both concerning outcome stratification and distribution of patients across risk groups.

Although some differences in MRD-based outcome stratification were noted between FLT3-ITD^−^ and FLT3-ITD^+^ patients for the individual time points pre-HCT, day 30, and day 100, MRD-low vs MRD-high patients showed improved outcomes at all peritransplant time points, irrespective of FLT3-ITD status (Figure 4B).

## Discussion

In summary, although stratification based on patient and treatment variables or MRD negativity alone had limited prognostic value, the application of optimized MRD thresholds and longitudinal integration of multiple measurements significantly improved our ability to predict post-HCT OS.

Prior analyses have reported superior outcome for patients with MRD negativity^4^, ^5^, ^6^^,^^8^ or reduction in *NPM1*-mutant transcript levels >3 or 4 log_10_ steps after intensive induction.^7^^,^^9^^,^^12^^,^^18^^,^^29^ At the pre-HCT time point, improved OS and reduced relapse risk were reported for MRD-negative patients^16^^,^^17^ and patients without detectable *NPM1*-mutant transcripts in archival samples analyzed by deep sequencing^23^^,^^30^ or droplet digital PCR.^15^ Furthermore, studies by Karas et al, Dillon et al, and Schwind et al explored the use of distinct thresholds (MRD <0.2% in PB and 1% in BM^14^; MRD <0.1% in BM^13^) to stratify patients in CR at the pre-HCT time point.^13^^,^^14^^,^^22^ An especially clear stratification of relapse rate and OS was obtained by *NPM1*-MRD droplet digital PCR pre-HCT measurements at a threshold of 0.01% and 1%, respectively, with no differences between the measurements in PB and BM,^22^ although with analyses performed on archival samples.

In contrast to the pre-HCT MRD analyses, the prognostic value of early post-HCT *NPM1*=MRD measurements and MRD thresholds vs longitudinal dynamics after HCT are largely underexplored. To our knowledge, only one study using RT-PCR analyzed the impact of *NPM1*=MRD early after HCT. Across 92 patients from multiple centers treated in prospective trials, MRD >1% in the first MRD assessment after HCT was associated with inferior survival, whereas patients with MRD <1% showed outcomes indistinguishable from MRD-negative patients. Further early analyses on mixed patient cohorts including post-HCT samples suggested prognostic significance for elevated *NPM1*-mutant transcript levels in postremission samples^6^ or increases >2 log_10_ steps in post-HCT follow-up samples.^26^ Additional studies showing an association of post-HCT MRD and outcomes are either limited by an extremely small sample size^24^ or employed deep sequencing instead of qRT-PCR,^23^^,^^25^ and a dedicated analysis of early post-HCT MRD has not yet been reported.

In our study, we decided to include all patients with *NPM1*-mutant AML who underwent a transplant at our centers, including those who underwent a transplant in persistent disease. Especially in the latter, MRD assessment at early post-HCT time points is of high interest due to their higher relapse risk. In patients receiving allo-HCT in CR, we confirmed predictive thresholds after HCT at comparable levels to the full data set. Recent findings by Fraccaroli et al, did not show significant differences between outcomes for MRD-negative and MRD-positive patients in CR pre-HCT.^21^ Consistently, the thresholding of pre-HCT MRD did not reveal a significant difference in OS among patients who underwent a transplant in CR1. Interestingly, our data suggest that thresholding is informative at all peritransplant time points in patients receiving allo-HCT in CR2 or with active disease, although subgroup sizes limit conclusions.

Given that MRD-negative and MRD-low patients show comparable OS, we suggest that increasing detection sensitivity (eg, by using BM vs PB or by using more sensitive assays) may not improve discrimination at peritransplant time points. Peritransplant *NPM1*-MRD may especially identify patients at high risk and may be less useful to delineate patients with excellent outcome, especially within the subgroup of patients treated with allo-HCT in CR and showing low or negative MRD before and after allo-HCT. Therefore, our data suggest that MRD assessment in BM may not add value beyond MRD assessment in PB at pre-HCT and day 30 post-HCT and cannot serve as a definitive assay to rule out MRD-high status in PB. This hypothesis should be taken into account and further prospectively evaluated, because from the patient perspective, BM assessments without clear additional benefit should be omitted.

Optimized outcome prediction with statistically derived thresholds between *NPM1*:*ABL1* ratios of 0.01% and 10% has been observed for patients at pre-HCT time points.^6^, ^7^, ^8^^,^^12^^,^^21^^,^^29^ In contrast, we demonstrate for the first time, to our knowledge, that thresholds significantly increase the predictive capacity of early post-HCT MRD assessments. MRD-low patients at day 30 and day 100 after HCT showed OS comparable with that of MRD-negative patients, whereas MRD-high patients had inferior outcomes. Conversely, we could not confirm an impact of FLT3-ITD on MRD-based outcome prediction, which has been reported in some studies,^14^ but not in other previous analyses.^21^^,^^22^ Going a step further, we show that multivariate peritransplant MRD risk scores based on longitudinal, thresholded MRD levels allow the clean stratification of long-term post-HCT OS and outperform single measurements and relative log-step changes between measurements among individual patients.

The absence of a prognostic impact of FLT3-ITD in our cohort likely reflects referral and treatment bias. Furthermore, definitions and clinical recommendations for the classification and management of FLT3-ITD^+^ *NPM1*-mutant AML changed over the duration of our study. These selection effects limit generalizability and should be addressed in a larger analysis by respective stratification, matching, and a more limited inclusion period.

In summary, we propose that thresholding is superior to the conventional interpretation of peritransplant MRD measurements and may aid in early post-HCT management decisions. The application of thresholding to peritransplant MRD provides a potentially generalizable distinction of MRD-high patients who should receive increased monitoring and, dependent on the time point, defined post-HCT interventions such as early reduction of immunosuppression or the application of maintenance/salvage treatments and/or donor lymphocyte infusions. Conversely, thresholding may help reduce morbidity caused by additional BM punctures and potential overtreatment of MRD-low patients.

For further multicenter validation, we are currently preparing a multicenter, real-world analysis of the prognostic impact of peritransplant MRD levels. This will determine whether peritransplant MRD thresholds may be generalized across assays and laboratories. In the next step, the safety and efficacy of applying such thresholds to clinical decision-making should be validated prospectively.

Conflict-of-interest disclosure: The authors declare no competing financial interests.
