# Supplementary material for: Predictive capacity of peritransplant measurable residual disease thresholds in NPM1-mutant acute myeloid leukemia
Source: Blood Adv. 2025 Dec 4;10(4):1082–93. doi: 10.1182/bloodadvances.2025017908 (PMC12915150; doi:10.1182/bloodadvances.2025017908)
Supplement: Supplemental Figures and Tables [file BLOODA_ADV-2025-017908-mmc1.pdf]

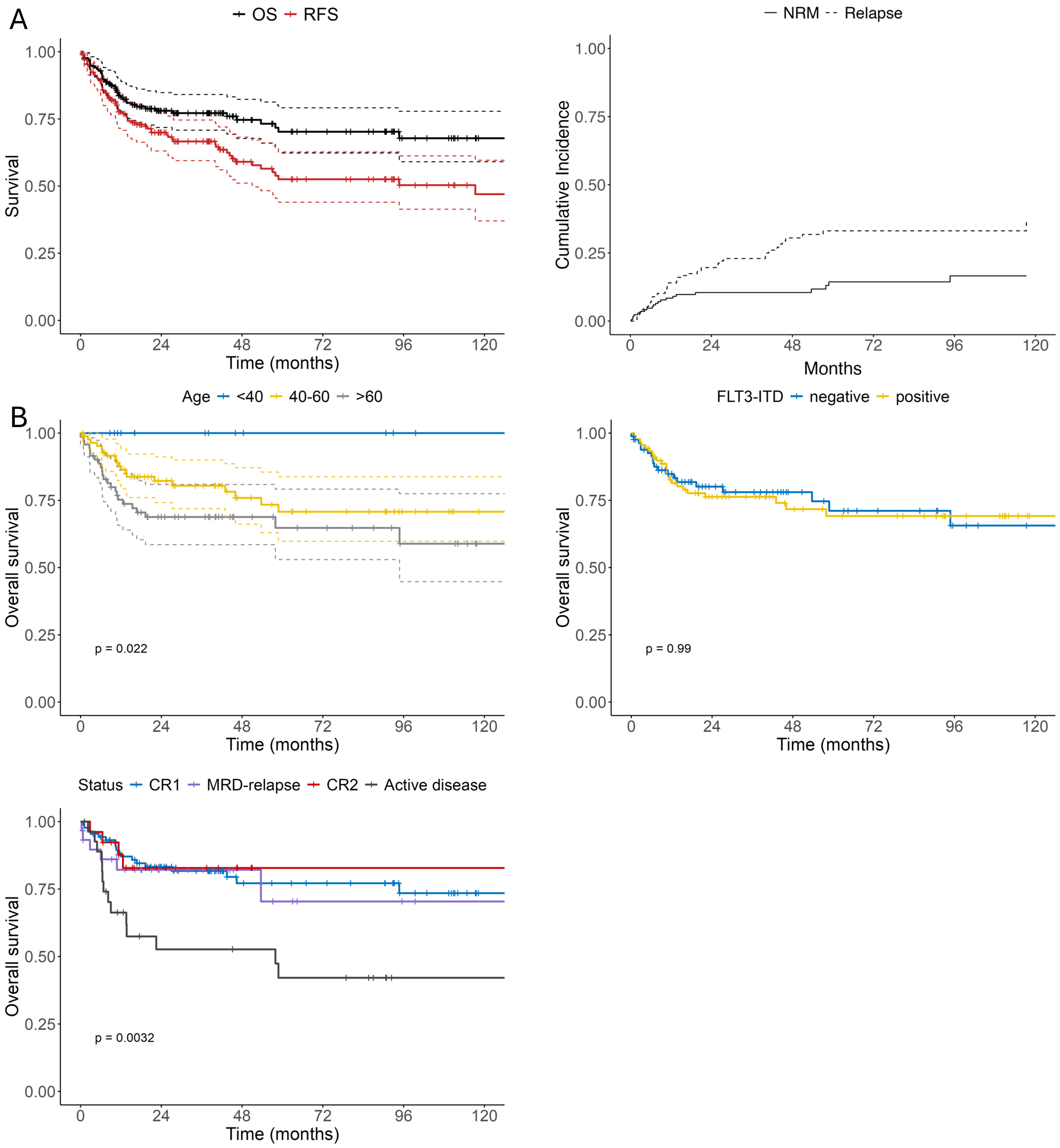

Figure S1: Overall alloHCT outcomes (A) and overall survival (OS) stratified by patient age, FLT3-ITD status, remission before alloHCT (B), N=172.

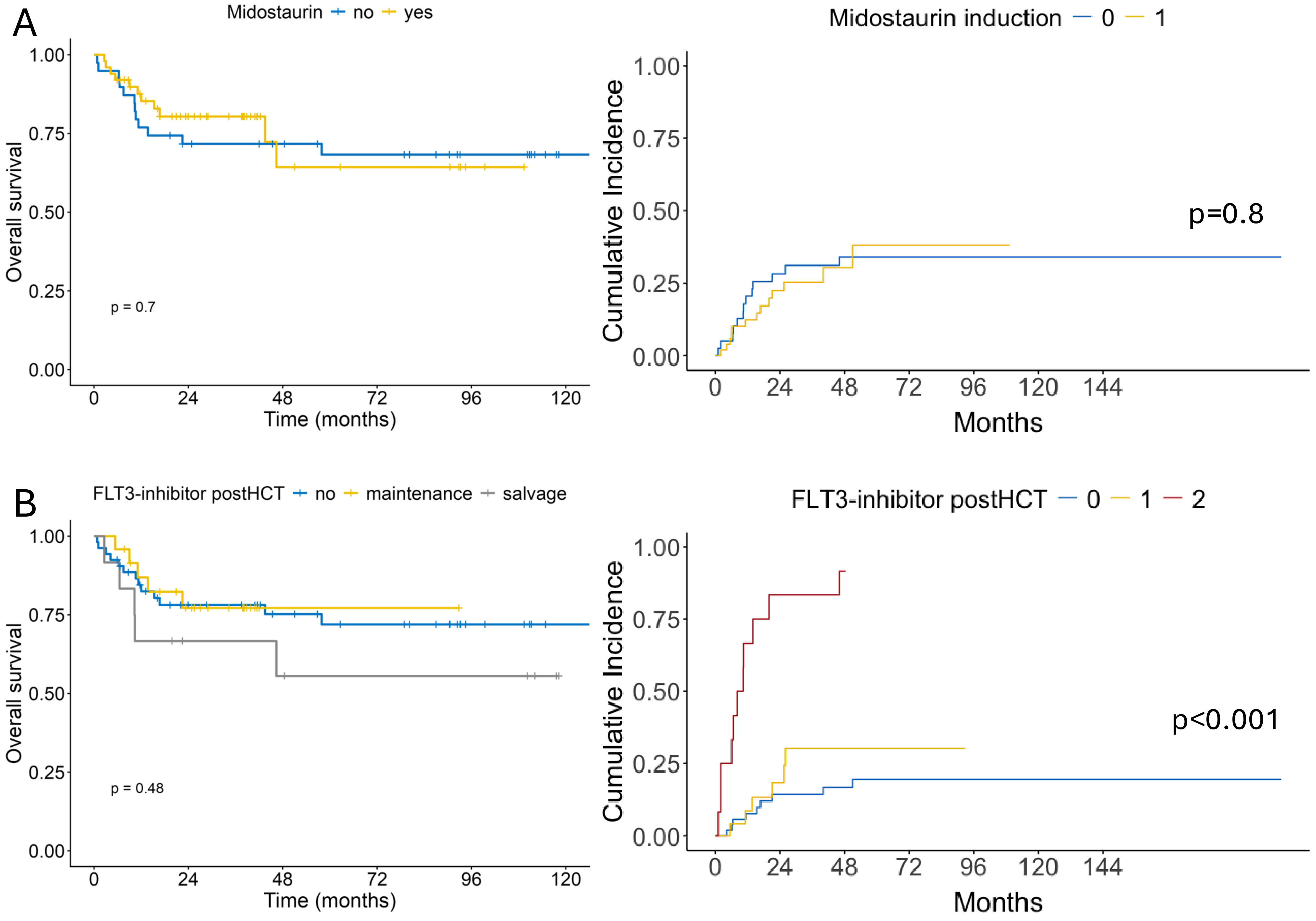

Figure S2: Overall survival (OS, left) and cumulative incidence of relapse (right) stratified by exposition to midostaurin prior to alloHCT during induction and/or consolidation in subgroup of all FLT3-ITD positive patients (A,) and stratified by exposition to FLT3-inhibitors after alloHCT as maintenance (1) or salvage treatment (2). n=89.

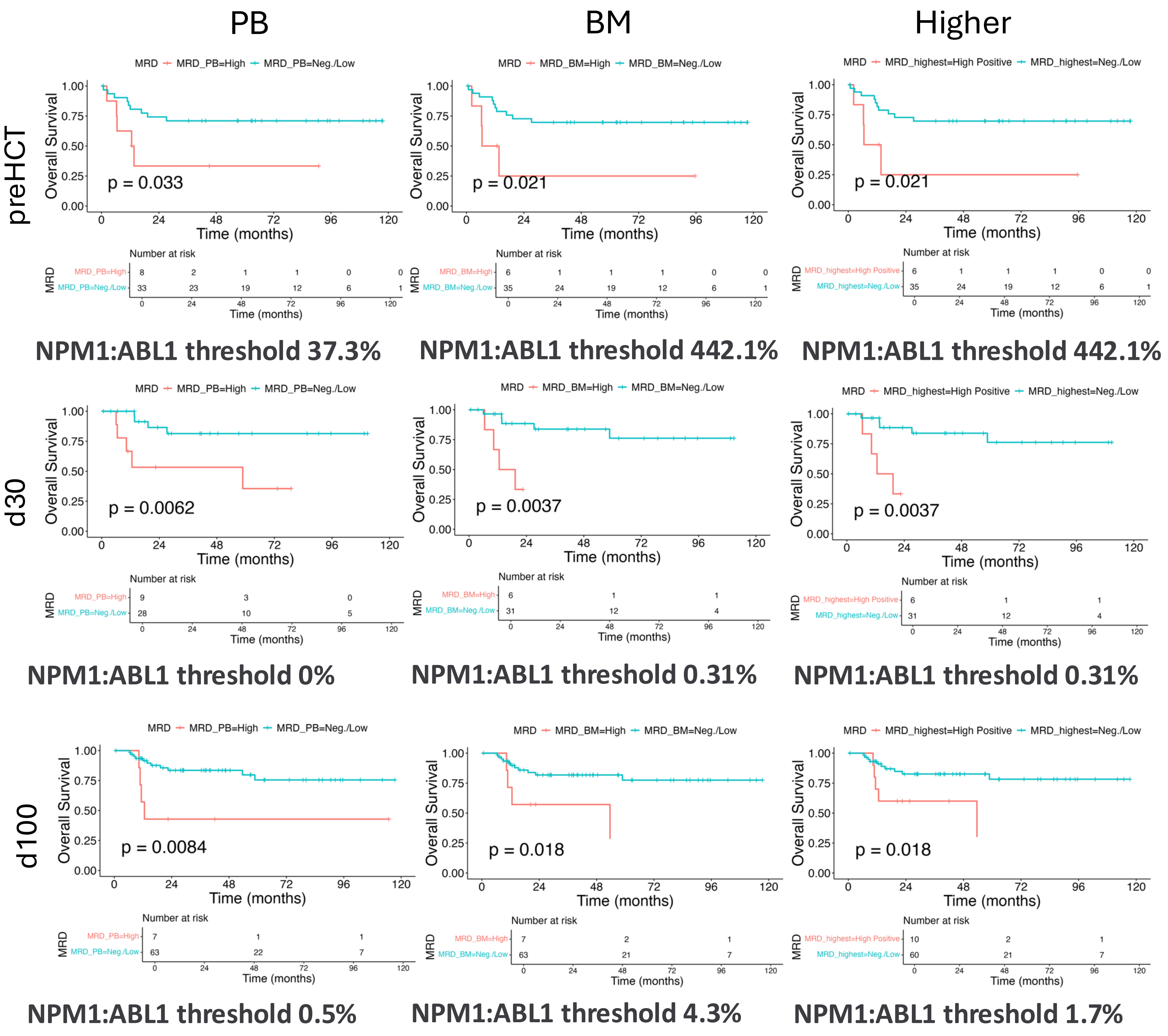

Figure S3: Head-to-head comparison of overall survival according to classification as MRD-high (above statistically derived threshold) or MRD-low (below statistically derived threshold) in either peripheral blood (PB) only, bone marrow (BM) only, according to the higher available value out of PB and BM at preHCT (n=41), d30 postHCT (n=37), or d100 postHCT (n=70). At each timepoint, thresholds are derived and applied to OS in the subset of patients with available MRD measurements from both PB and BM.

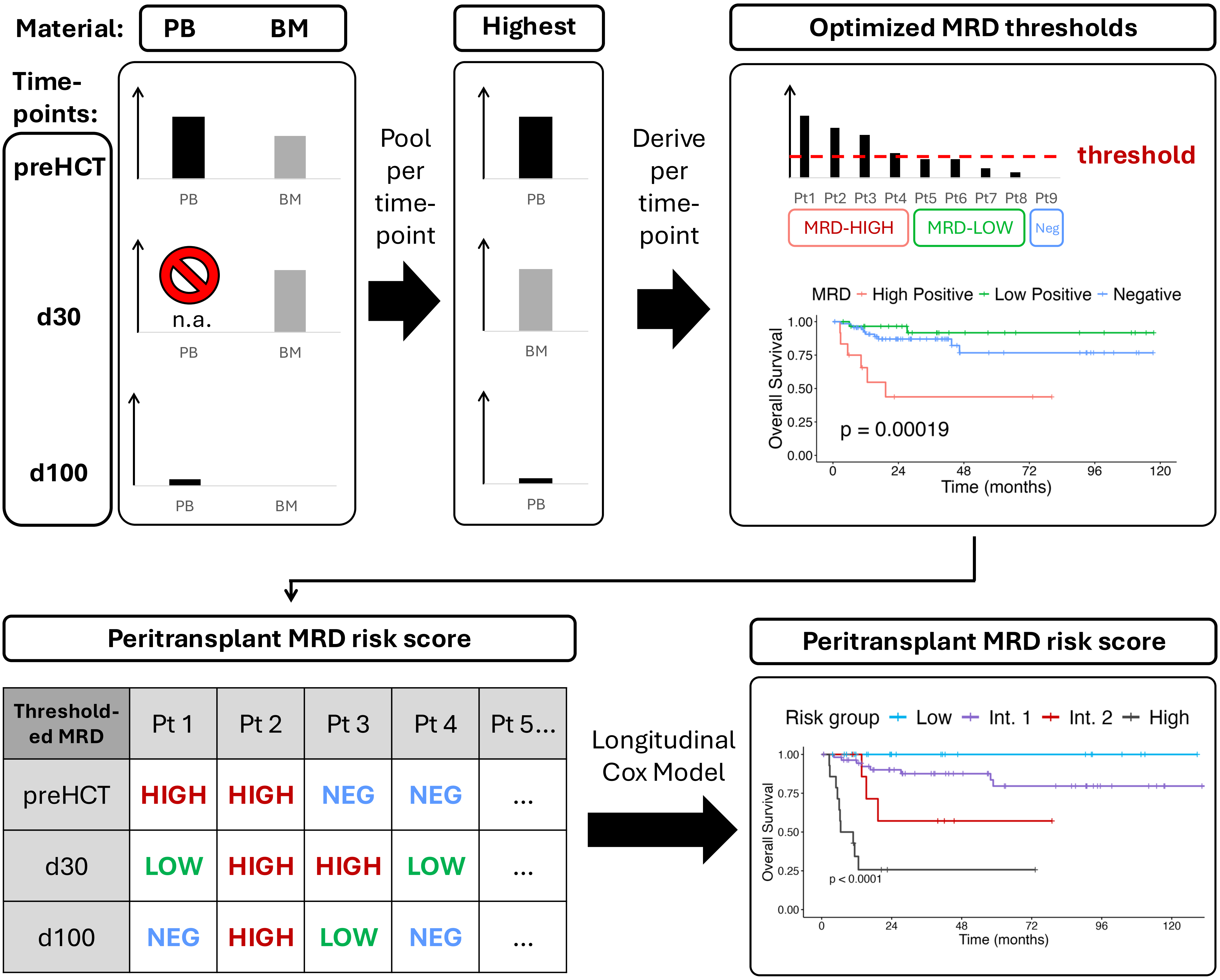

Figure S4: Implementation of MRD-thresholding and peritransplant MRD risk score.
First, available MRD measurements in peripheral blood (PB) or bone marrow (BM) at the peritransplant timepoints preHCT, d30 postHCT, and d100 postHCT are pooled. If at one timepoint measurements from both PB and BM are available, the higher value is selected.
Next, using maximally selected rank statistics, MRD thresholds optimized to distinguish patients with good or poor overall survival (OS) are derived for each of the three timepoints across patients. Based on these thresholds, each patient is classified as MRD-high (above threshold), MRD-low (positive, but below threshold), or MRD-negative at each of the three timepoints. Last, a longitudinal multivariate Cox model is generated from this MRD-classification at all three or only two of the peritransplant assessment timepoints. This model can be used to calculate a peritransplant MRD risk score and assign patients to prognostic risk groups low, intermediate (int.) 1, int. 2, and high to optimally stratify overall survival.

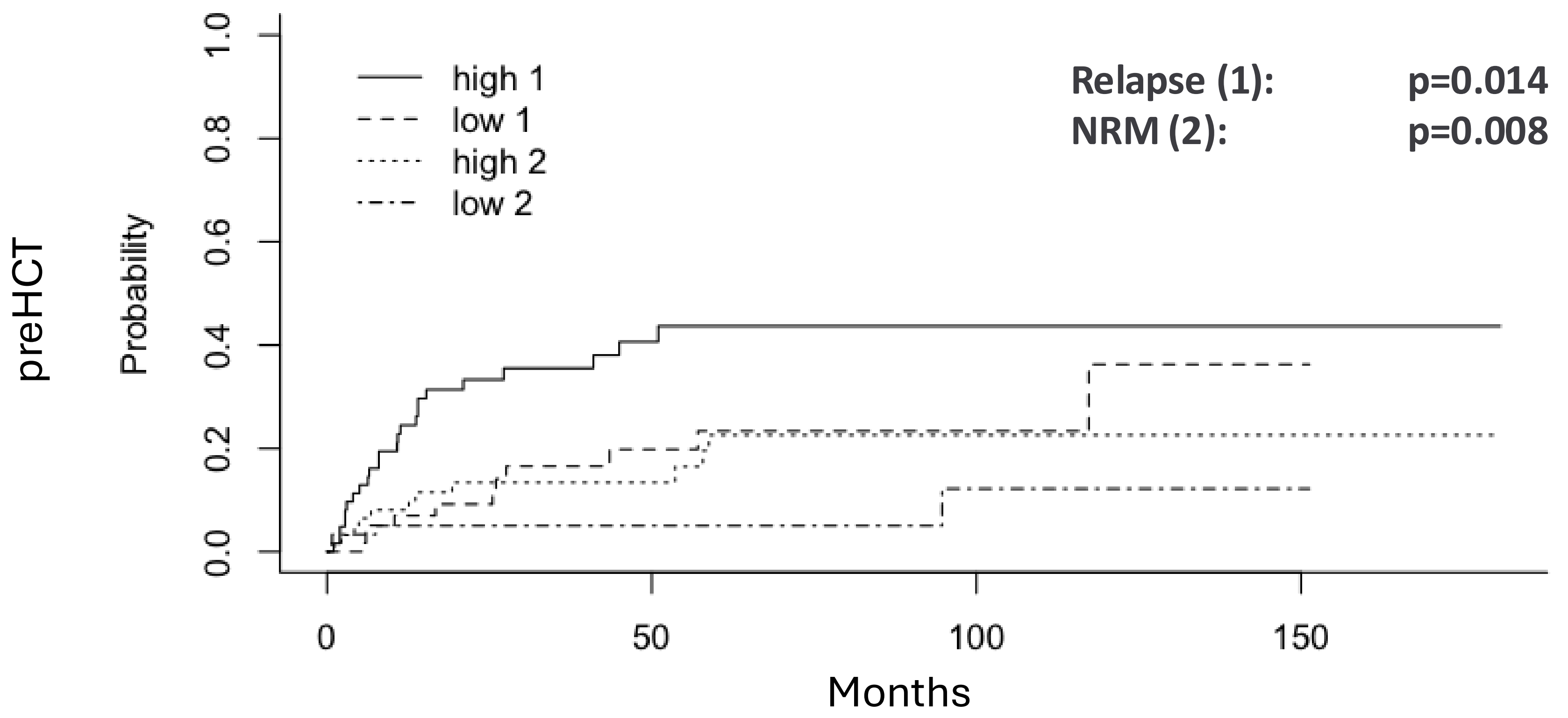

NPM1:ABL1 threshold: Relapse (1) 0.63, NRM (2) 83.0

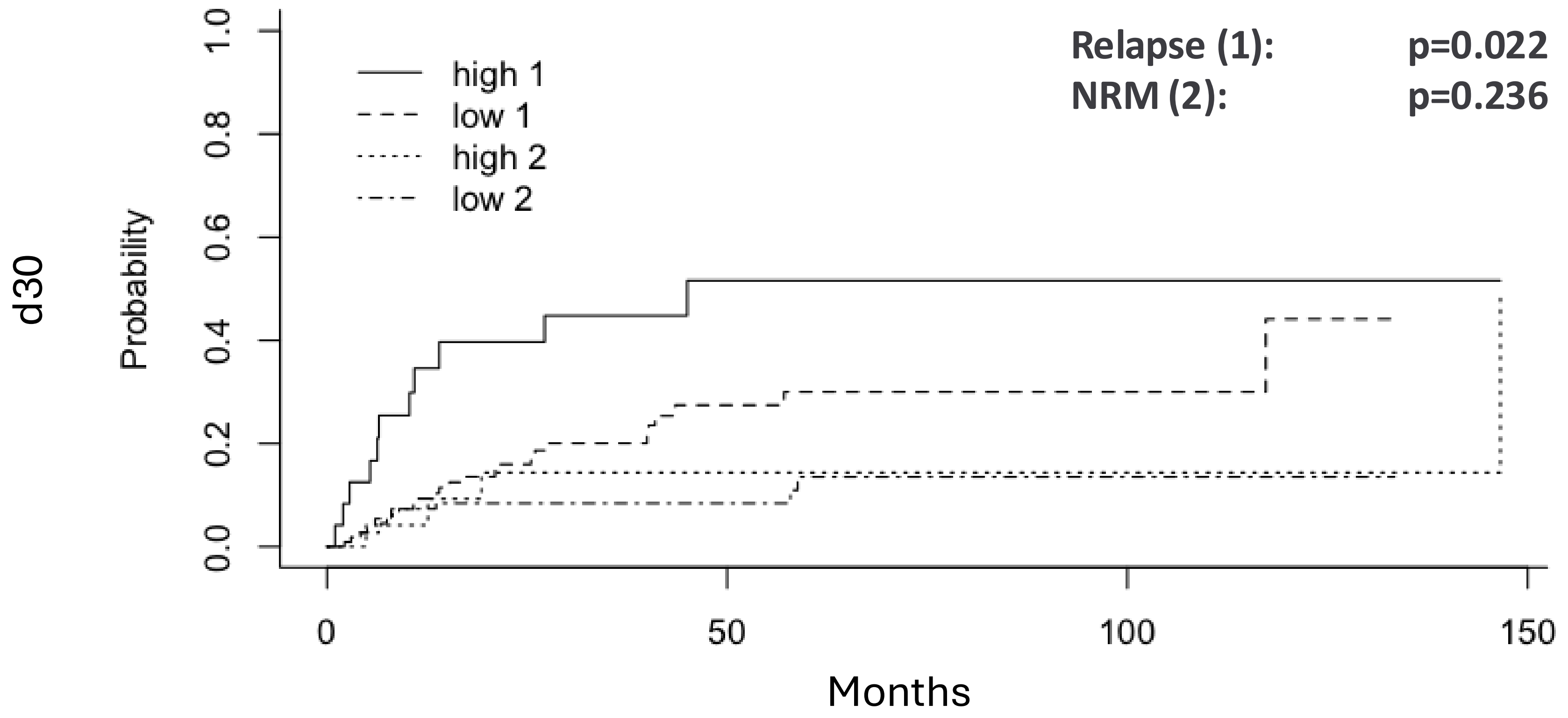

NPM1:ABL1 threshold: Relapse (1) 0.09, NRM (2) 0.08

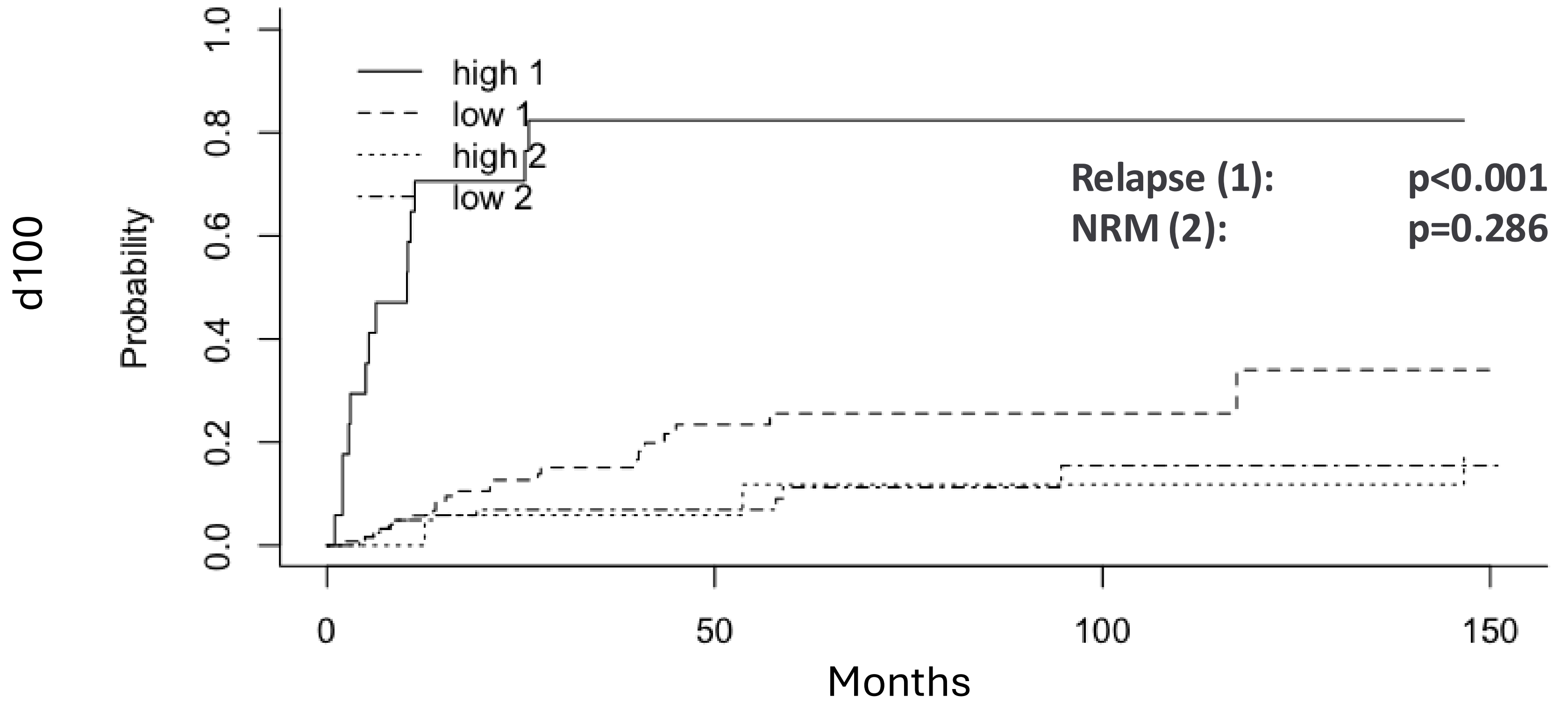

NPM1:ABL1 threshold: Relapse (1) 2.25, NRM (2) 0.08

Figure S5: Competing-risks analysis of cumulative incidence of relapse (lines with label 1) and non-relapse mortality (lines with label 2). Maximally selected rank statistics were used to identify thresholds to optimize stratification of relapse incidence and NRM based on MRD measurements above (high) or below (low) these thresholds at peritransplant timepoints preHCT (n=124), d30 postHCT (n=137), and d100 postHCT (n=144). Differences between MRD-high and MRD-low groups were tested for significance using Gray's test.

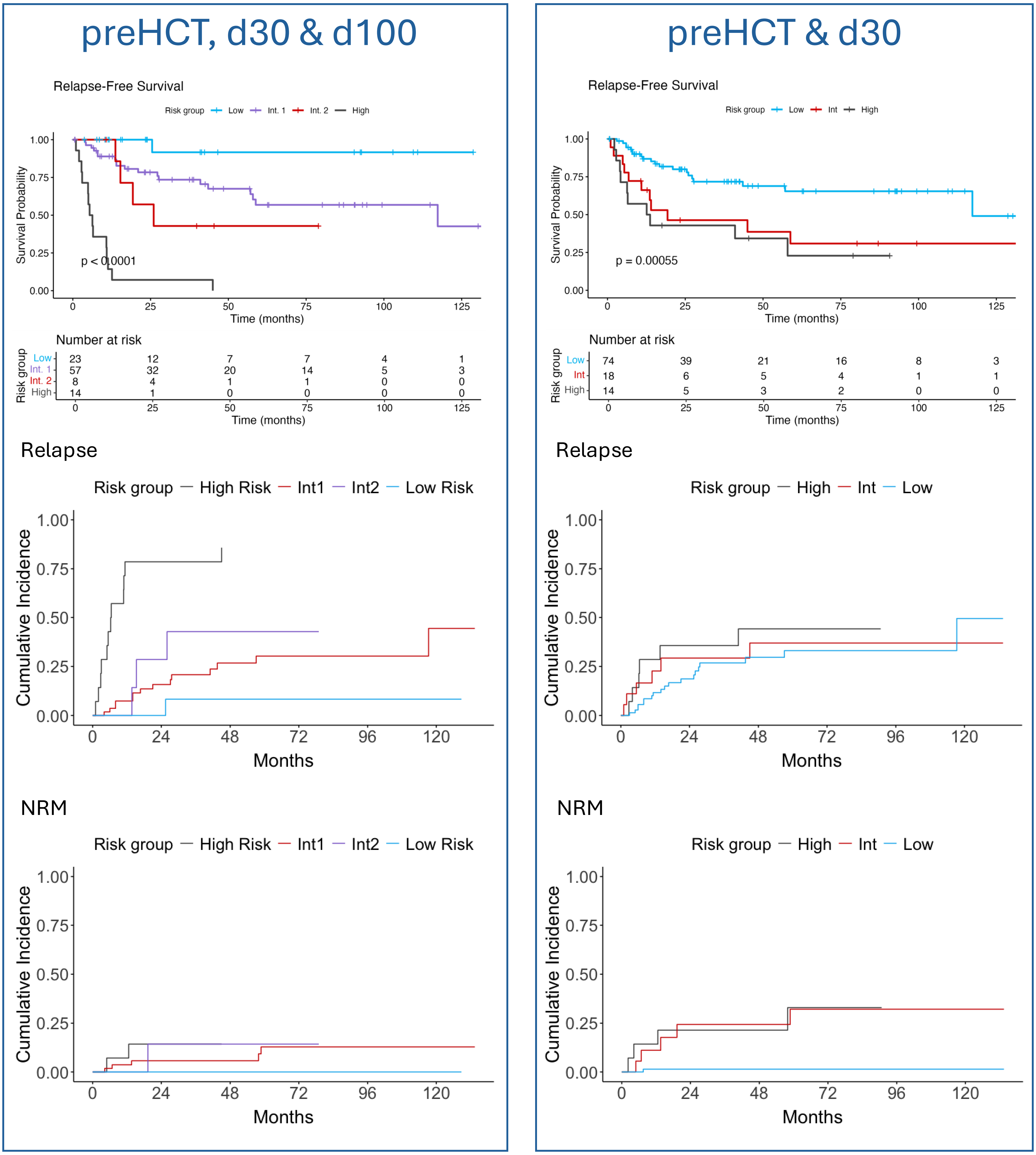

Figure S6: Multivariate Cox proportional Hazards regression models were fitted using MRD-levels stratified into MRD-negative, MRD-low, MRD-high at peritransplant timepoints preHCT, d30 and d100 (plots to the left) or preHCT and d30 only (plots to the right). MRD tresholds and optimal substrata were statistically derived for optimized prediction of overall survival (OS). The graphs compare relapse-free survival (RFS) , relapse incidence, and non-relapse mortality (NRM) between risk groups that optimally stratify OS. For NRM and relapse, death and relapse were treated as competing events.

A

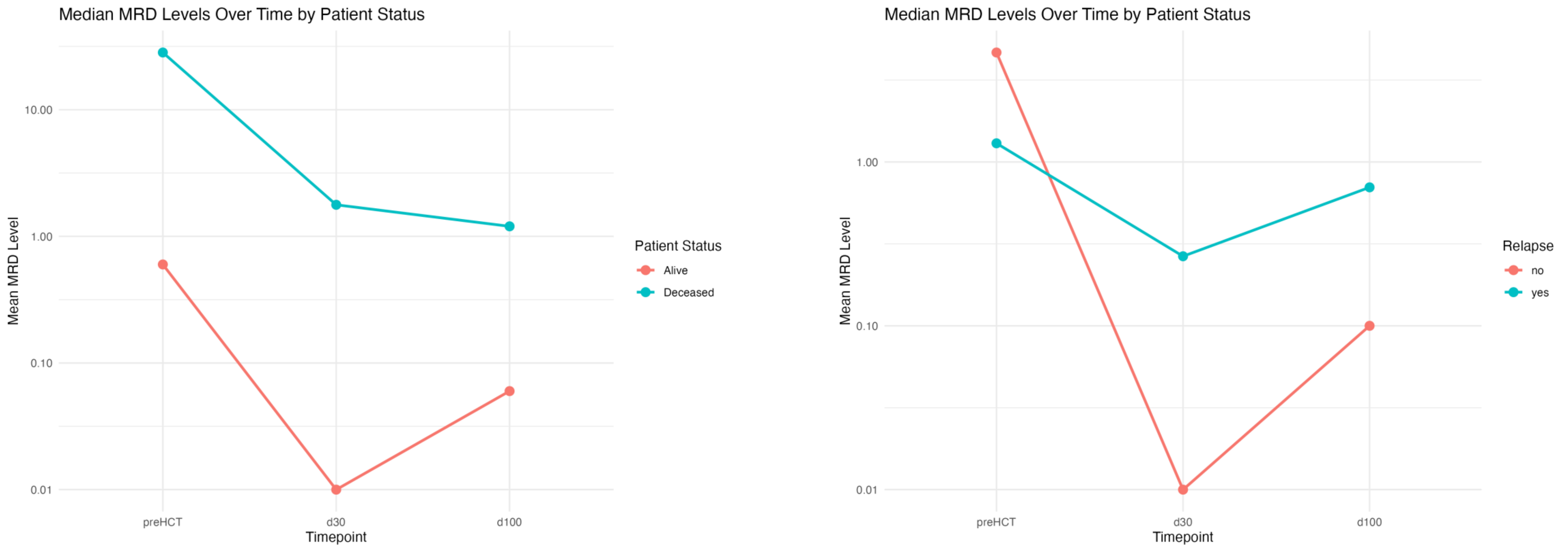

B

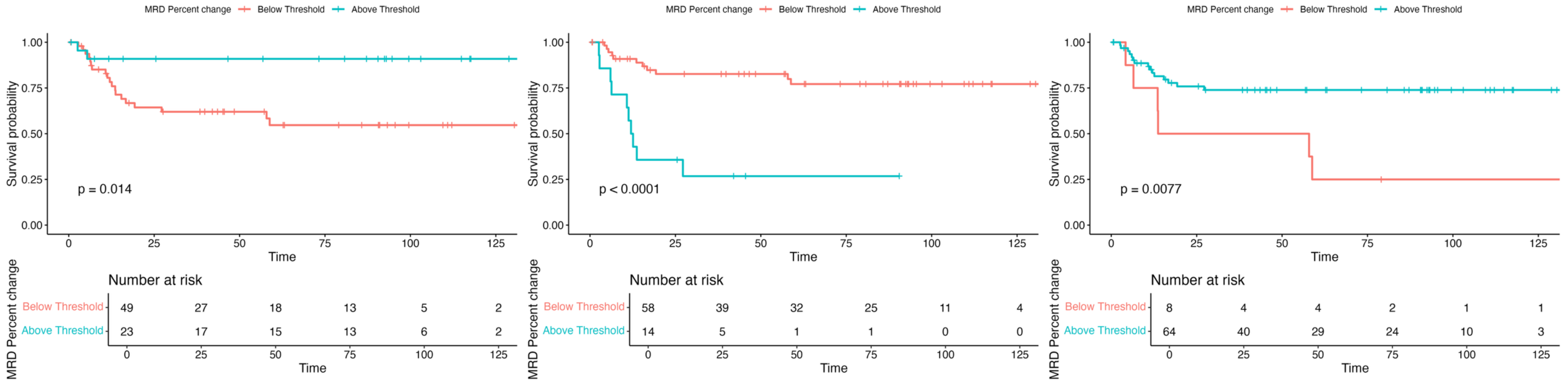

Figure S8:
A: Median MRD levels at and changes between preHCT, d30 and d100 postHCT timepoints in patients with postHCT death of any cause (left) or relapse (right) during follow-up. Analysis in 115 patients from Tuebingen cohort with available MRD at individual timepoints, n=93 (preHCT), n=82 (d30), n=89 (d100).
B: Percent changes between MRD levels at the timepoints preHCT and d30 postHCT (left), d30 and d100 (center), and preHCT and d100 (right) were calculated. For these relative changes, optimized thresholds for OS stratification were derived using maximized rank statistics: -97.4% (~1.6 log_{10} reduction) for preHCT to d30; 1900% (~1.3 log_{10} increase) for d30 to d100; -99.99998% (~6.7 log_{10} reduction) for preHCT to d100. Shown is the comparison of OS according to classification of the relative change as below threshold (i.e., relative change smaller than or more negative than threshold) or above threshold (i.e., relative change greater than or less negative than threshold) using Kaplan-Meier estimator and log-rank test. Analysis in n=72 patients from Tuebingen cohort with available MRD at all three timepoints.

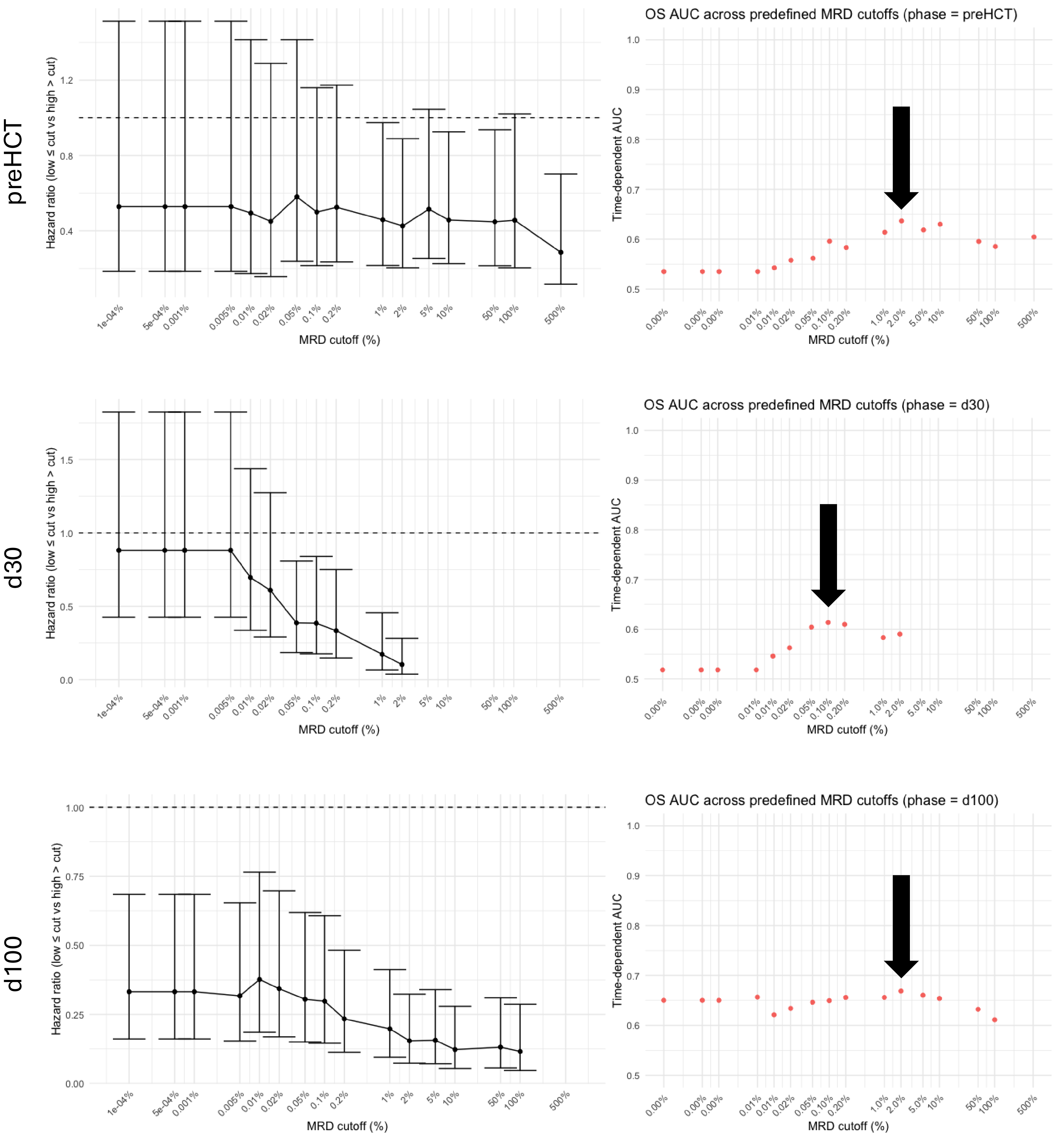

Figure S9:
Univariate hazard ratios (HRs) ± 95% confidence interval for overall survival (OS) in MRD-low vs MRD-high patients (left panels) and area under the receiver operator curve (AUROC) for MRD-based OS prediction up to 24 months post alloHCT plotted across a range of clinically useful absolute MRD-tresholds (0.0001, 0.0005, 0.001, 0.005, 0.01, 0.02, 0.05, 0.1, 0.2, 1, 2, 5, 10, 50, 100, and 500 percent) to separate MRD-high and –low patients. Analysis was performed for peritransplant timepoints preHCT, and d30 and d100 postHCT. Vertical arrows indicate MRD thresholds with highest AUROC for OS prediction.

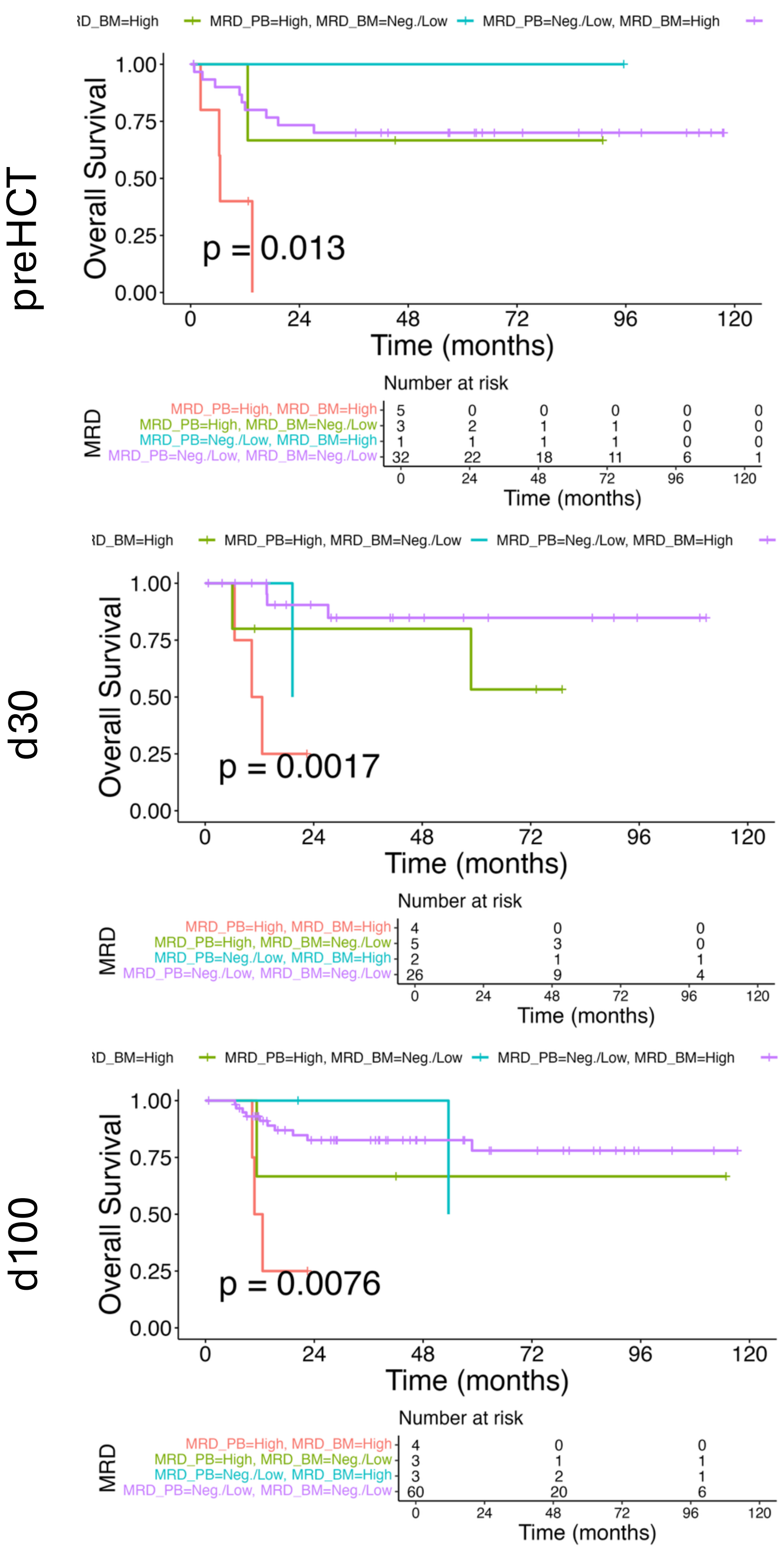

Figure S10:
Overall survival according to combination of thresholded NPM1 MRD in peripheral blood (PB) and bone marrow (BM) at peritransplant timepoints preHCT, d30, and d100. The graphs show the possible combinations of a patient being either MRD-low/negative or MRD-high in PB and BM at each timepoint.

Conditioning intensity	Conditioning regimen	N	%
MAC	Busulfan/Cyclophosphamide	10	9%
	Cyclophosphamide/TBI 12 Gy	7	6%
RIC	Fludarabine/Busulfan	22	19%
	Fludarabine/Treosulfan	31	27%
	Fludarabine/BCNU/Melphalan	4	3%
	Fludarabine/Thiotepa/Melphal	3	3%
	not specified	1	1%
	Fludarabine/TBI 2-8 Gy	16	14%
Sequential RIC	FLAMSA	17	15%
	HD-Mel	1	1%

Table S1: Types of applied conditioning regimens for n=115 patients with available data.

	Strata	n (%)	n total	OS - HR (univariable)
Age	continuous		172	1.06 (1.03-1.10, **p<0.001**)
Sex	male	80 (46.5)	172	-
	female	92 (53.5)		1.24 (0.67-2.27, p=0.491)
Karnofsky	100	39 (34.8)	112	-
	<=90	73 (65.2)		2.16 (0.99-4.75, p=0.054)
FLT3-ITD	no	83 (48.3)	172	-
	yes	89 (51.7)		1.00 (0.55-1.82, p=0.990)
Midostaurin during induction/consolidation	no	39 (43.8)	89	-
	yes	50 (56.2)		0.85 (0.37-1.96, p=0.705)
FLT3-inhibitor post alloHCT	none	53 (59.6)	89	-
	as maintenance	24 (27.0)		0.96 (0.34-2.72, p=0.932)
	as salvage treatment	12 (13.5)		1.80 (0.64-5.06, p=0.264)
Number of inductions	1	43 (37.4)	115	-
	2	72 (62.6)		1.18 (0.59-2.37, p=0.637)
Number of consolidations	continuous		115	1.21 (0.98-1.49, p=0.079)
Status HCT	CR1	88 (51.2)	172	-
	CR1 with MRD relapse	30 (17.4)		1.22 (0.48-3.08, p=0.673)
	CR2	26 (15.1)		0.90 (0.30-2.66, p=0.845)
	no CR	28 (16.3)		3.18 (1.59-6.35, **p=0.001**)
Conditioning	RIC	98 (85.2)	115	-
	MAC	17 (14.8)		0.22 (0.05-0.94, **p=0.041**)
Donor Mismatch	No	96 (83.5)	115	-
	Yes	19 (16.5)		2.60 (1.24-5.42, **p=0.011**)

Table S2: Results of univariate Cox regression analysis for association between patient characteristics, treatment variables and overall survival (OS), shown as hazard ratios (HR), 95% confidence intervals (CI) and univariate p-values.

	preHCT	preHCT %	d30	d30 %	d100	d100 %	All timepoints	All %
**PB**	58	33.7%	76	44.2%	102	59.3%	39	22.7%
**BM**	107	62.2%	98	57.0%	112	65.1%	43	25.0%
**pooled**	124	72.1%	137	79.7%	144	83.7%	102	59.3%

Table S3: Availability of MRD-measurements in peripheral blood (PB), bone marrow (BM) or one of both at timepoints preHCT, d30 and d100 postHCT, and all timepoints, in absolute numbers and percentage of total patients (n=172).

Timepoint	n	Spearman's rho	Spearman p value	C_index_PB	C_index_PB_BM	delta_C_index	AUROC_PB	AUROC_PB_BM	delta_AUROC
preHCT	41	0.834	<0.0001	0.582	0.598	0.016	0.543	0.546	0.002
d30	37	0.78	0.0026	0.721	0.705	-0.015	0.802	0.829	0.027
d100	70	0.831	<0.0001	0.646	0.684	0.038	0.654	0.711	0.057

Table S4: Correlation of NPM1 MRD measured in peripheral blood (PB) versus bone marrow (BM) was analyzed using Spearman's test at the three timepoints preHCT, and d30 and d100 postHCT. Analysis of incremental value of including BM MRD for outcome prediction in subset of patients with MRD measured in both PB and BM available at each timepoint was performed by generating Cox regression models including only MRD in PB vs. models including MRD in PB and BM at the three timepoints preHCT, d30 and d100. For both models, the C index and AUROC to predict OS up to 24 months post alloHCT were calculated and the delta analyzed.

preHCT	n	threshold	HR	CI_lower	CI_upper	p value	C index	AUROC
PB	41	37.34	3.16	1.04	9.60	0.043	0.58	0.54
BM	41	442.10	3.71	1.13	12.16	0.031	0.48	0.52
highest	41	442.10	3.71	1.13	12.16	0.031	0.52	0.49
Pooled highest	124	1.63	2.56	1.20	5.44	0.014	0.64	0.62
**d30**	**n**	**threshold**	**HR**	**CI_lower**	**CI_upper**	**p value**	**C index**	**AUROC**
PB	37	0.00	5.28	1.40	19.88	0.014	0.72	0.80
BM	37	0.31	5.82	1.52	22.22	0.010	0.64	0.60
highest	37	0.31	5.82	1.52	22.22	0.010	0.67	0.66
Pooled highest	137	0.11	3.26	1.49	7.11	0.003	0.59	0.59
**d100**	**n**	**threshold**	**HR**	**CI_lower**	**CI_upper**	**p value**	**C index**	**AUROC**
PB	70	0.50	4.18	1.32	13.27	0.015	0.65	0.65
BM	70	4.30	3.66	1.16	11.56	0.027	0.65	0.67
highest	70	1.70	3.42	1.16	10.09	0.026	0.63	0.65
Pooled highest	144	1.70	6.48	3.09	13.58	<0.001	0.72	0.75

Table S5: Maximized rank statistics were used to statistically derive optimized MRD tresholds (NPM1:ABL1 ratio in %) at the timepoints preHCT, d30 postHCT, or d100 postHCT, using either peripheral blood (PB) only, bone marrow (BM) only, or the higher available value out of PB and BM. For head-to-head comparison, the three approaches were directly compared in the subset of patients with MRD out of both PB and BM available at each timepoint (rows PB, BM, highest). In addition, patients with availability of either only PB or only BM at a timepoint were included and pooled with patients with availibility of both PB and BM using the higher available value in the latter (pooled highest).
Predictive capacity of each approach (material combination and respective threshold) was evaluated using univariate Cox regression. Results are displayed as hazard ratios (HR) for patients with MRD-high vs. MRD-low, 95% confidence intervals (CI) and univariate p-values. Overall correlation between MRD values and overall survival (OS) up to 5 years after alloHCT were assessed using area under the receiver operator curve (AUROC). All 172 patients were included, lower numbers per timepoint result from missing measurements in individual patients at some of the timepoints.

Timepoint	median threshold	threshold_IQR_lo	threshold_IQR_hi	HR_median	HR_IQR_lo	HR_IQR_hi	C_index_mean	C_index_sd	iAUC_mean	iAUC_sd
preHCT	7.55	0.56	79.61	1.62	1.18	2.32	0.561	0.049	0.553	0.057
d30	0.11	0.077	0.3	3.10	2.02	4.84	0.604	0.060	0.613	0.073
d100	0.83	0.3	1.7	5.03	3.02	7.83	0.657	0.065	0.686	0.080
Longitudinal model global							0.783	0.078	0.815	0.081

Table S6: Using bootstrap resampling (200 replicates) with out-of-bag validation, we assessed the stability and performance of maximally selected rank cutoffs for the pooled highest MRD measurements at each of the timepoints preHCT, d30 and d100. The table shows the median ± interquartile range (IQR) of the MRD thresholds, median of resulting hazard ratios (HR) between MRD-high and –low, mean C-index ± standard deviation (sd) of the model and mean AUROC ± sd to predict overall survival up to 24 months post alloHCT. In addition, the bootstrapping was performed for the global C-index and AUROC of the longitudinal model consisting of tresholded MRD levels preHCT, d30 and d100.

	Stratum	Low Risk	Int 1	Int 2	High Risk	p
Total N (%)		23 (22.5)	57 (55.9)	8 (7.8)	14 (13.7)	
Age at diagnosis	Mean (SD)	50.0 (8.9)	55.2 (12.0)	57.2 (9.0)	61.0 (8.0)	**0.023**
Age at diagnosis	<40	3 (13.0)	6 (10.5)	0 (0.0)	0 (0.0)	**0.045**
	40-60	17 (73.9)	27 (47.4)	5 (62.5)	5 (35.7)	
	>=60	3 (13.0)	24 (42.1)	3 (37.5)	9 (64.3)	
Sex	male	9 (39.1)	33 (57.9)	5 (62.5)	7 (50.0)	0.462
	female	14 (60.9)	24 (42.1)	3 (37.5)	7 (50.0)	
FLT3 ITD	negative	9 (39.1)	32 (56.1)	2 (25.0)	8 (57.1)	0.247
	positive	14 (60.9)	25 (43.9)	6 (75.0)	6 (42.9)	
Status HCT	CR1	17 (73.9)	40 (70.2)	6 (75.0)	5 (35.7)	**0.012**
	CR2	5 (21.7)	4 (7.0)	0 (0.0)	5 (35.7)	
	no CR	1 (4.3)	13 (22.8)	2 (25.0)	4 (28.6)	
Midostaurin during induction/consolidation	No	4 (28.6)	11 (44.0)	4 (66.7)	4 (66.7)	0.299
	Yes	10 (71.4)	14 (56.0)	2 (33.3)	2 (33.3)	
FLT3-inhibitor post alloHCT	none	8 (57.1)	19 (76.0)	3 (50.0)	2 (33.3)	**0.019**
	as maintenance	6 (42.9)	3 (12.0)	3 (50.0)	1 (16.7)	
	as salvage	0 (0.0)	3 (12.0)	0 (0.0)	3 (50.0)	

Table S7: Patient characteristics and treatment variables according to peritransplant MRD risk score subgroup low, intermediate (int) 1, int 2, or high risk. Fisher's exact test was used to test for differences in the composition of substrata between groups. Information on conditioning intensity, donor match and Karnofsky performance index omitted because only available for 115 out of 172 patients.
